# An Unusual Presentation of Moyamoya Disease in a Pediatric Patient

**DOI:** 10.7759/cureus.61804

**Published:** 2024-06-06

**Authors:** Ola Shahrour, Abdulqader Al Zubaidi, Salwa Al Kaabi

**Affiliations:** 1 Department of Academic Affairs, Tawam Hospital, Al Ain, ARE; 2 Emergency Department/Pediatric Emergency, Sheikh Shakhbout Medical City, Abu Dhabi, ARE; 3 Department of Pediatrics, Tawam Hospital, Al Ain, ARE

**Keywords:** revascularization surgery, pediatric stroke, cerebral ischemia, cerebral angiography, moyamoya disease

## Abstract

Moyamoya disease (MMD) is a chronic cerebro-vasculopathy that is extremely rare in the pediatric population. The main characteristic feature is the progressive stenosis in the internal carotid artery with or without the involvement of its main branches in the circle of Willis leading to ischemic stroke. Patients have clinical manifestations related to cerebral ischemia in the carotid branch territories, such as sensory impairment, hemiparesis, and aphasia/dysarthria. Herein, we report a case of MMD in a six-year-old Emirati female who presented with unusual manifestations of MMD in the form of headache, vomiting, and double vision and was diagnosed with MMD based on a brain MRI with angiography. To our knowledge, this is the first reported case of MMD in the United Arab Emirates.

## Introduction

Acute stroke is rare in the pediatric population. Moyamoya disease (MMD) is a rare cerebrovascular disease of unknown etiology. It is characterized by a chronic non-atherosclerotic occlusive cerebrovascular disease that leads to progressive steno-occlusion of the intracranial part of the internal carotid arteries and the proximal part of their branches, with abnormal formation of collateral vessels [1-3]. First described as "hypoplasia of bilateral internal carotid arteries" by Takeuchi in 1957, the term "Moyamoya" was described by Suzuki et al. in 1969, which describes the imaging of the collateral vessels that compensate for progressive stenosis showing a "puff of smoke" [2]. The highest incidence of MMD is reported in Japan. The crude prevalence rate in 2003 was estimated to be “6.03 per 100,000 population” in Japan and it is incidence is 0.54 per 100,000 with a male-to-female ratio of 1:1.8 with female predominance [4,5]. We report a six-year-old female presented with unusual manifestations of MMD in the form of headache, vomiting, and double vision and was diagnosed to have MMD based on brain magnetic resonance imaging (MRI) with angiography.

This case report was previously presented as a poster at the “18th SEHA International Pediatric Conference” on February 17, 2024.

## Case presentation

A six-year-old previously healthy girl presented to the emergency department (ED) for the first time with a history of intermittent, progressive, and severe early morning headache, with nonprojectile vomiting and double vision for a one-year duration. The family denied any history of head trauma, fever, ear discharge, weakness, abnormal gait, seizures, changes in her personality, changes in her level of consciousness, numbness, dizziness, photophobia, phonophobia, neck pain, neck stiffness, easy bruises, or bleeding tendency in the past. Her past histories were unremarkable, including normal developmental milestones, and her family history was negative for any neurological disease, heart disease, sickle cell disease, neurofibromatosis, developmental delay, seizure disorders, genetic disorders, or autoimmune diseases.

On examination, the patient was alert and oriented with a Glasgow Coma Scale score of 15/15. Vitals signs were normal, except for high blood pressure readings (114/76), which is considered stage 1 hypertension. In a neurological examination, there were no signs of meningeal irritation, no neurocutaneous markers, and no asymmetry of the face. Her gait was normal, and the tone was slightly increased in the left upper extremity with normal tone in the right upper and both lower extremities. Her power was 5/5 in all extremities. Deep tendon reflexes were normal, and Babinski sign was normal with no clonus and negative cerebellar signs except for dysmetria. The cranial nerve examination was unremarkable. The ophthalmic examination showed no papilledema and no visual field defect. All other systems examinations were found to be normal.

Clinical and laboratory evaluation excluded secondary causes of the vascular changes, including hematologic, cardiac, renal, metabolic, toxicological causes, or any evidence of systemic vasculitis (Table 1).

**Table 1:** Laboratory investigation results showing normal results, excluding secondary causes of the vascular changes, including hematologic, metabolic, toxicological causes, or any evidence of systemic vasculitis. WBC: white blood cell; HB: hemoglobin; PT: prothrombin time; PTT: partial thromboplastin time; INR: international normalized ratio; LDL: low-density lipoprotein; HDL: high-density lipoprotein; TSH: thyroid-stimulating hormone; ANA: anti-nuclear antibody; ENA: extractable nuclear antigen; IgG: immunoglobulin G; IgM: immunoglobulin M; P-ANCA: perinuclear anti-neutrophil cytoplasmic antibodies; C-ANCA: cytoplasmic-antineutrophil cytoplasmic autoantibody; CRP: C-reactive protein; ESR: erythrocyte sedimentation rate.

Laboratory test	Result	Reference range
Hematology		
WBC	7.6 x 10^9/L	5-14.5 x 10^9/L
Hemoglobin	14.3 g/L	11.5-14.5 g/L
Platelets	451 x 10^9/L	140-400 x 10^9/L
Hemoglobin electrophoresis		
Hb A	96.70%	95-98%
Hb A2	2.90%	2-3%
Hb F	0.40%	<2%
Coagulation panel		
PT	11.4 sec	11.7-15.1 sec
PTT	27 sec	31.8-43.7 sec
INR	1.05	0.87-1.2
Protein C	78.60%	68.3-143.5%
Protein S	87.60%	61-105%
D-dimer	<0.19 mg/L	0.12-0.53 mg/L
Anti-thrombin III	100%	81-133%
Factor V	0.97	0.81-1.13
Fibrinogen	2.52 g/L	1.5-3.8 g/L
Metabolic		
Amino acid profile	Normal	-
Urine organic acid	Normal	-
Homocysteine level	Normal	-
Lipid profile		
Total cholesterol	2.78 mmol/L	2.8-4.8 mmol/L
LDL	1.69 mmol/L	<2.59 mmol/L
HDL	0.76 mmol/L	0.9-2.1 mmol/L
Triglyceride	0.73 mmol/L	0.25-085 mmol/L
TSH	1.41 milli IU/L	0.6-4.8 milli IU/L
Immunological		
ANA	Negative	-
ENA	Negative	-
C3	1.38 g/L	0.88-1.55 g/L
C4	0.33 g/L	0.12-0.32 g/L
Cardiolipin IgG	Negative	-
Cardiolipin IgM	Negative	-
P-ANCA	Negative	-
C-ANCA	Negative	-
CRP	<2 mg/L	0.1-1 mg/L
ESR	2 mm/h	0-10 mm/h
Urine toxicology screen	Negative	-

**Figure 1:**
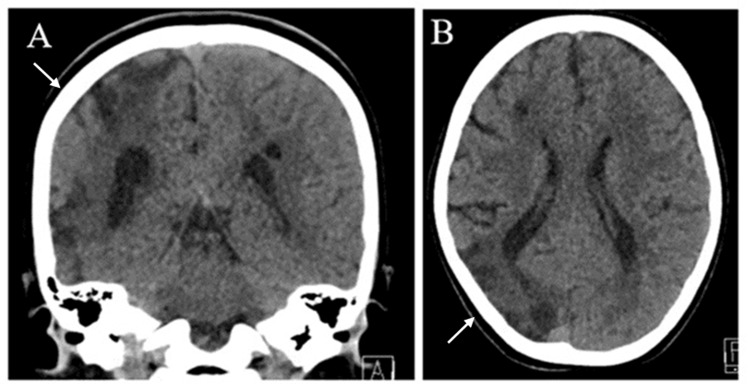
Brain computed tomography showing right parieto-occipital hypodensity, with loss of grey-white matter differentiation and loss of volume. Bilateral subcortical hypodensities in the right frontal and left parietal regions. The overall image suggests an old ischemic insult. (A) Coronal view. (B) Sagittal view. The arrows point to the areas of parieto-occipital hypodensity, with loss of grey-white matter differentiation and loss of volume.

A computed tomography (CT) scan showed right parieto-occipital hypodensity, with loss of grey-white matter differentiation and loss of volume suggestive of old ischemic insult (Figure 1).

**Figure 2:**
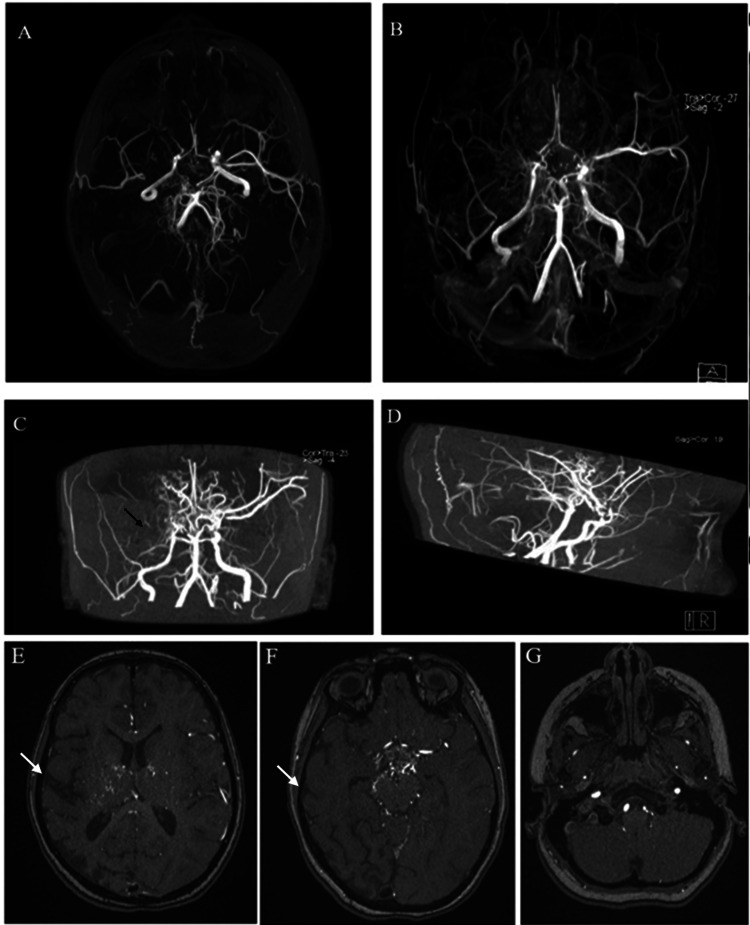
(A) MRA showing narrowing and occlusion of the internal carotid artery and middle cerebral artery with collateral vessels showing the “puff of smoke” appearance. (B) Transverse view. (C) Coronal view. (D) Sagittal view. (E-G) MRV showing areas of encephalomalacia in the right parietal and occipital lobe with few bilateral old lacunar infarcts associated with subacute ischemic infarction involving the left parietal lobe. The white arrows point to the areas of encephalomalacia in the right parietal and occipital lobe in the MRV. The black arrow points to the narrowing and occlusion of the internal carotid artery and middle cerebral artery. MRA: magnetic resonance angiography; MRV: magnetic resonance venography.

MRI with magnetic resonance angiography (MRA) and magnetic resonance venography (MRV) of the brain was suggestive of MMD (Figure 2).

There were significant abnormal net-like proliferating vessels/collaterals at the level of the circle of Wills in the suprasellar cistern giving the characteristic puff of smoke appearance. In addition, there were multiple tortuous flow voids on T1 and T2-weighted images involving the lenticulostriate, thalamo perforating, leptomeningeal, and dural arteries on both sides, which was suggestive of MMD.

She was treated conservatively with aspirin 5 mg/kg/day and referred to a specialized center for surgical intervention where cerebral revascularization surgery using encephaloduroarteriosynangiosis (EDAS) was done. In the follow-up evaluation nine months after the surgery, the patient showed improvement, and her neurological examination was normal.

## Discussion

MMD is a rare disease in the pediatric population that is characterized by a narrowing in the cerebral circulation associated with an unusual network of collateral blood vessels resembling a net. "Moyamoya" is a Japanese word that represents the description of the angiography imaging of cerebral vessels that appears like a "puff of smoke" [1,2].

Although initially believed to be prevalent only in Japan, cases have now been reported worldwide, with a higher frequency in Asia and other non-Caucasian regions [3,5]. The occurrence and prevalence of MMD in the United Arab Emirates (UAE) is not yet determined. To our knowledge, this is the first case of MMD to be reported in the UAE.

As per the reported cases, MMD has been observed to have a bimodal age distribution. One peak occurs at the age of five, and a second, lower peak is seen at around 40 years old [3]. However, a more recent study indicated a shift in this pattern, with the highest peak now occurring between 45 and 49 years, and a secondary peak between five and nine years, which suggests that the incidence of pediatric MMD is declining [5].

The exact cause of MMD remains unknown, but recent articles and studies have shed light on associated genetic mutations and the pathophysiology of this rare disease [5-8]. The narrowing of cerebral blood vessels appears to be a response of the brain's vasculature to various external stimuli, injuries, or genetic abnormalities. MMD has been found to be associated with conditions such as sickle cell anemia, neurofibromatosis type 1, Down syndrome, congenital heart defects, antiphospholipid syndrome, renal artery stenosis, and thyroiditis. Nevertheless, it is important to note that over half of the affected children have no identifiable cause for their MMD [6,9]. In our case, no association with any systemic disease was described.

In pediatric cases, the prevailing clinical presentation of MMD involves ischemic events (accounting for 80% of cases) and is more prevalent than hemorrhagic presentations, usually leading to symptoms like mono or hemiparesis, sensory impairment, or aphasia/dysarthria. In rarer instances, patients may experience atypical symptoms, such as syncope, visual disturbances, or pseudo-psychiatric symptoms. Interestingly, some patients with MMD may also report a headache resembling migraine-like characteristics [10,11]. A review article proposed that this type of headache could be attributed to the stimulation of dural nociceptors caused by dilated transdural collaterals. Furthermore, the gradual and progressive narrowing of intracranial blood vessels can result in hypoperfusion leading to an intracranial oligemic state, which has been identified as a possible cause of the headache as well [10].

In the indexed case, the patient presented with headache, vomiting, and visual disturbances, which emphasizes the significance of including MMD as a potential diagnosis when evaluating pediatric patients with headaches. Typically, MRA scans are utilized to validate the diagnosis; however, cerebral angiography is the gold standard for diagnosis. Imaging helps to observe the vascular anatomy involved by showing regions of infarctions and visualize narrowing and occlusions in the cerebral vessels and extensive collateral flow through the perforating vessels, displaying the characteristic appearance akin to a "puff of smoke." The narrowing of blood vessels and reduced blood flow tend to worsen over time despite medical interventions unless surgical treatment is provided [7]. In the acute phase, the primary approach of management usually focuses on lowering intracranial pressure, enhancing cerebral blood flow, and managing seizures. Currently, revascularization procedures are performed to increase blood flow to the oxygen-deprived brain tissue. Existing literature supports the effectiveness of these procedures, with reported positive outcomes in terms of symptom improvement across all age groups in the long term [3,10].

The prognosis of MMD varies and is influenced by age and the type of presentation. After surgical procedures, hemodynamic improvement appears to be consistent across all age groups. Additionally, cases presenting with transient ischemic attacks (TIAs) and epileptiform clinical patterns tend to have a more favorable long-term outcome compared to cases involving infarctions [3,12].

## Conclusions

MMD is a rare disease in the pediatric population that is manifested by significant narrowing of cerebral vessels leading to ischemic changes that are presented by acute, transient, or permanent hemiparesis and sensory impairment. In rare instances, it may present with atypical symptoms such as syncope, visual disturbances, or pseudo-psychiatric symptoms. Our case draws attention to a significant challenge faced in the clinical setting, given the disease's exceptional rarity among the UAE population, coupled with the atypical clinical manifestations observed, and the significance of early diagnosis and management for favorable patient outcomes.
